# Integrating multi-platform genomic datasets for kidney renal clear cell carcinoma subtyping using stacked denoising autoencoders

**DOI:** 10.1038/s41598-019-53048-x

**Published:** 2019-11-13

**Authors:** Tongjun Gu, Xiwu Zhao

**Affiliations:** 1 Bioinformatics, Interdisciplinary Center for Biotechnology Research, University of Florida, Gainesville, FL USA; 2 Department of Ophthalmology & Visual Sciences, University of Michigan, Ann Arbor, MI USA

**Keywords:** Cancer genomics, Tumour biomarkers, Renal cell carcinoma, Data integration, Machine learning

## Abstract

Clear cell renal cell carcinoma (ccRCC) is highly heterogeneous and is the most lethal cancer of all urologic cancers. We developed an unsupervised deep learning method, stacked denoising autoencoders (SdA), by integrating multi-platform genomic data for subtyping ccRCC with the goal of assisting diagnosis, personalized treatments and prognosis. We successfully found two subtypes of ccRCC using five genomics datasets for Kidney Renal Clear Cell Carcinoma (KIRC) from The Cancer Genome Atlas (TCGA). Correlation analysis between the last reconstructed input and the original input data showed that all the five types of genomic data positively contribute to the identification of the subtypes. The first subtype of patients had significantly lower survival probability, higher grade on neoplasm histology and higher stage on pathology than the other subtype of patients. Furthermore, we identified a set of genes, proteins and miRNAs that were differential expressed (DE) between the two subtypes. The function annotation of the DE genes from pathway analysis matches the clinical features. Importantly, we applied the model learned from KIRC as a pre-trained model to two independent datasets from TCGA, Lung Adenocarcinoma (LUAD) dataset and Low Grade Glioma (LGG), and the model stratified the LUAD and LGG patients into clinical associated subtypes. The successful application of our method to independent groups of patients supports that the SdA method and the model learned from KIRC are effective on subtyping cancer patients and most likely can be used on other similar tasks. We supplied the source code and the models to assist similar studies at https://github.com/tjgu/cancer_subtyping.

## Introduction

Tailoring treatments to specific groups of patients becomes very popular in recent years due to the confirmation that the fundamental biology of each patient is unique, such as DNA, RNA, protein and methylation. Recent studies have shown that tumor is a highly heterogeneous tissue. With progression, the tumor becomes more and more divergent, which is a leading challenge to the treatments. Therefore, stratifying the patient is an initial and important step for developing personalized treatments.

RCC is one of the top 10 most common cancers in the world^1^. There are three major histologic subtypes of RCC from a clinical viewpoint^2^: clear cell renal cell carcinoma, papillary RCC, and chromophobic RCC. ccRCC is not only the most common subtype, account for 80–90% of all RCC cases^2^, but also the major cause of the deaths from kidney cancer^1^. Recent studies demonstrated that ccRCC is highly heterogeneous either intra-tumor or inter-tumor^3,4^. ccRCC is classified into four grades (1–4)^5^ according to Fuhrman grading system. Grade 1 and 2 can be classified as low grade, and grade 3 and grade 4 are high grades but behaves differently on 5-year cancer-specific survival^5^. ccRCC also has four stages (1–4) according to American Joint Committee on Cancer staging^6^. The tumor at Stage I and II is still local. At stage III, the tumor extends to major veins or spreads to the perinephric tissues but is not beyond Gerota’s fascia. At Stage IV, the tumor migrates to distant metastasis. According to the staging system, different treatments were suggested for different stages of cancer. For localized cancer, partial nephrectomy approach is generally higher recommended than radical Nephrectomy^2^. When tumor migrates to other locations, systematic therapy is recommended^2^. In recent years, increased genomic and molecular studies on ccRCC greatly aided the diagnosis and treatments. Such as, the target therapy, mTOR pathway inhibitor, is benefit from genetics studies^7,8^. However, most of these studies focused on genetics variants discovery or identifying biomarkers for subtypes of RCC. In our study, we developed a new method that can integrate multi-platform genomic datasets to subtype ccRCC.

With the fast increase of huge amount of multi-platform datasets, more and more computational methods have been developed to identify the characteristics of each cancer patient. However, most methods are applied on one type of data or study each type of data separately. K-means clustering and Principal Component Analysis (PCA) are commonly used to classify the genomic data. Although both methods work well on one type of datasets, they have not been applied directly to cross-platform datasets. The TCGA network developed an algorithm called Cluster of Cluster (COC), re-clustering the clusters by converting the initial clusters computed from each platform into binary vectors^9^. Except the COC method, Bayesian method^10^ and non-negative matrix factorization method^11^ have also been developed to integrate datasets from multi-platform to do clustering. However, few of them are designed to handle very complicated multi-modality datasets from both intra-platform and inter-platform. In recent years, several deep learning methods have been developed to solve the difficulties on analyzing the complicated multi-platform datasets^12,13^. All of them are based on stacked Restricted Boltzmann Machines (sRBM), a generative algorithm, which uses RBM to capture the features of the data from each platform first and then join the latent variables from each platform to generate the common features of the cross-platform datasets. Authors showed that the sRBM successfully grouped the patients into clinically relevant subtypes. The successful application of the sRBM method on cancer patient classification inspired us to develop another deep learning method, autoencoder, a discriminative model, for cancer patient classification. Since a generative algorithm is unlikely to accurately model complicated datasets, a discriminative algorithm may be better on classification for complicated datasets.

In this work, we developed a stacked denoising autoencoder (SdA) to integrate gene expression, protein expression, microRNA (miRNA) expression, methylation and CNA datasets to subdivide the KIRC patients. We successfully divided the patients into two groups, in one of which had significantly higher survival probability than the other. We further identified a set of genes, miRNAs and proteins from differential analysis between the two groups, which can be potential biomarkers for diagnosis and developing new proper treatments. In comparison with K-means clustering method, the results showed that the SdA method produced a much clearer separation of the patients based on the clinical output. We also compared with stacked of Restricted Boltzmann Machines (sRBM) method and obtained consistent results. To further validate our model and show its generalization, we applied the learned KIRC model as a pre-trained model to independent datasets, LUAD and LGG datasets, and classified LUAD and LGG into clinical associated subtypes. Using the learned model as a pre-trained model can save the computing time on selecting the best model parameters and can improve the model from learning directly from the original dataset. We supplied both the learned models and the source codes for usages on similar tasks at https://github.com/tjgu/cancer_subtyping.

## Results

### The SdA model

**Figure 1:**
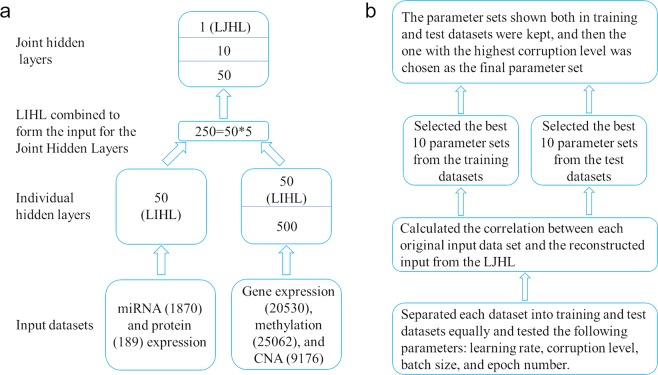
The model diagram of deep learning methods. (**a**) The layer structure of the SdA and sRBM methods. LJHL represents the Last Joint Hidden Layer. LIHL represents the Last Individual Hidden Layer. The numbers in the figure are the hidden units in the respective hidden layer. (**b**) The flowchart of the SdA model parameter selection process.

There are much less miRNAs (1870) and proteins (189) than genes (20530), methylations (25062) and CNAs (9176). Therefore, we used one hidden layer with 50 units for miRNA and protein datasets, but two hidden layers with 500 and 50 units separately for gene expression, methylation and CNA datasets. We named the last hidden layer for each dataset as the Last Individual Hidden Layer (LIHL) (Fig. 1a). We combined the LIHL from the five datasets, which resulted in 250 hidden units. Then three joint hidden layers were built with 50, 10, and 1 unit/units for each layer. We labeled the last joint hidden layer as the Last Joint Hidden Layer (LJHL). We trained each layer separately to obtain the optimal output for the input of the next layer. The KIRC patients were subtyped into two groups using K-means based on the hidden values from the LJHL, namely KIRC_SdA_G1 and KIRC_SdA_G2. The model was depicted in Fig. 1a. To obtain the best model, we tested all the combinations of several parameters in a wide range using two-fold cross validation: the learning rate at 0.001, 0.005, 0.01, 0.05, 0.1, 0.15, 0.2, 0.3 and 0.5; the corruption level at 0.01, 0.05, 0.1, 0.2 and 0.5; the batch size at 10, 20, 50 and 100; and the number of epoch at 1000, 2000 and 5000 (Fig. 1b). We took the correlation between the input data and the reconstructed input from the LJHL as a criterion to select the model parameters, which was independent of the cross entropy used in the training process. We added the correlations from each dataset and obtained two summed correlations for the training and test datasets respectively. We selected 10 sets of parameters for the training and test datasets based on the top 10 highest correlations produced between the input datasets and the reconstructed input from the LJHL. Then the sets of parameters shown in both the training and test datasets were kept. Finally, the set of parameters with the highest corruption level was chosen as the best parameter set for the classification, which was leaning rate at 0.01, corruption level at 0.2, batch size at 50 and the epoch at 5000. The process was shown in Fig. 1b. The subtypes for each patient was shown in Supplementary Table 1.

### Correlations between the original input and the reconstructed input from the LIHL and LJHL

**Figure 2:**
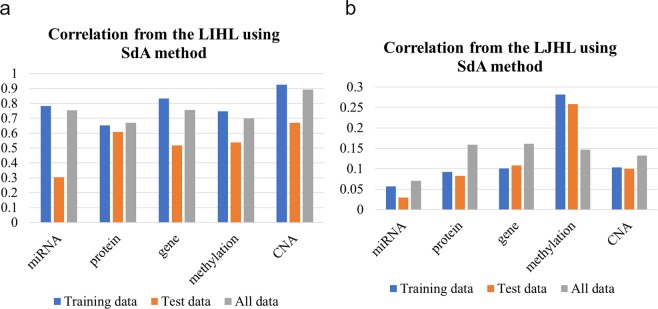
Pearson correlation between the KIRC original input and the reconstructed input from the LIHL and LJHL for the SdA method. (**a**) The correlation from the LIHL. (**b**) The correlation from the LJHL. Y axis is the correlation and x axis is the five datasets: miRNA expression, protein expression, gene expression, methylation and CNA. We split each dataset into two subsets: training and test datasets. All data represents the combination of the training and test datasets.

We did Pearson correlation analysis between the reconstructed input from the LIHL with each input dataset. The training datasets and the majority of the test datasets have correlations higher than 0.5 although the training datasets had higher correlations than the test datasets (Fig. 2a), demonstrating that the hidden variables in our model represent intrinsic features of our input datasets.

We also did Pearson correlation analysis between the reconstructed input from the LJHL with each original input. All the correlations were positive with the lowest at 0.029 for miRNAs in the test dataset, the highest at 0.281 for methylations in the training dataset (Fig. 2b). The results indicate that the final joint hidden variables contributed positively to the reconstruction.

Finally, similar correlation analysis was done on the whole dataset (combining the training and test datasets), more consistent results across the five datasets were observed both with LJHL and LIHL (Fig. 2a,b), indicating better consistent results can be obtained when the sample size of datasets was increased.

### The association of each subtype of patients with clinical features

In our work, we cannot assess the classification results based on the labels since we do not have the labels for the patients. Nevertheless, the goal of classifying patients is to identify the genomic features to assist the diagnosis and personalized treatments. Therefore, the better we can classify the patients into clearer clinical associated groups, the better the method works. We used this criterion to evaluate different methods on classification. And three clinical associated features were used in our study: the patient survival time, neoplasm histologic grade and pathologic stage. As described in the introduction, there are generally four neoplasm histologic grades and four pathologic stages for tumors, and the lower grade and the lower stage of the tumor is closer to a normal tissue. Grade 4 (G4) is the highest grade that the tumor has the fastest growth and spreading. The tumor at Stage II is larger than Stage I, but is still local. At Stage III, tumor has grown into the vein and may be lymph node. Stage IV is the highest stage that the tumor spreads into distant parts of the body.

**Figure 3:**
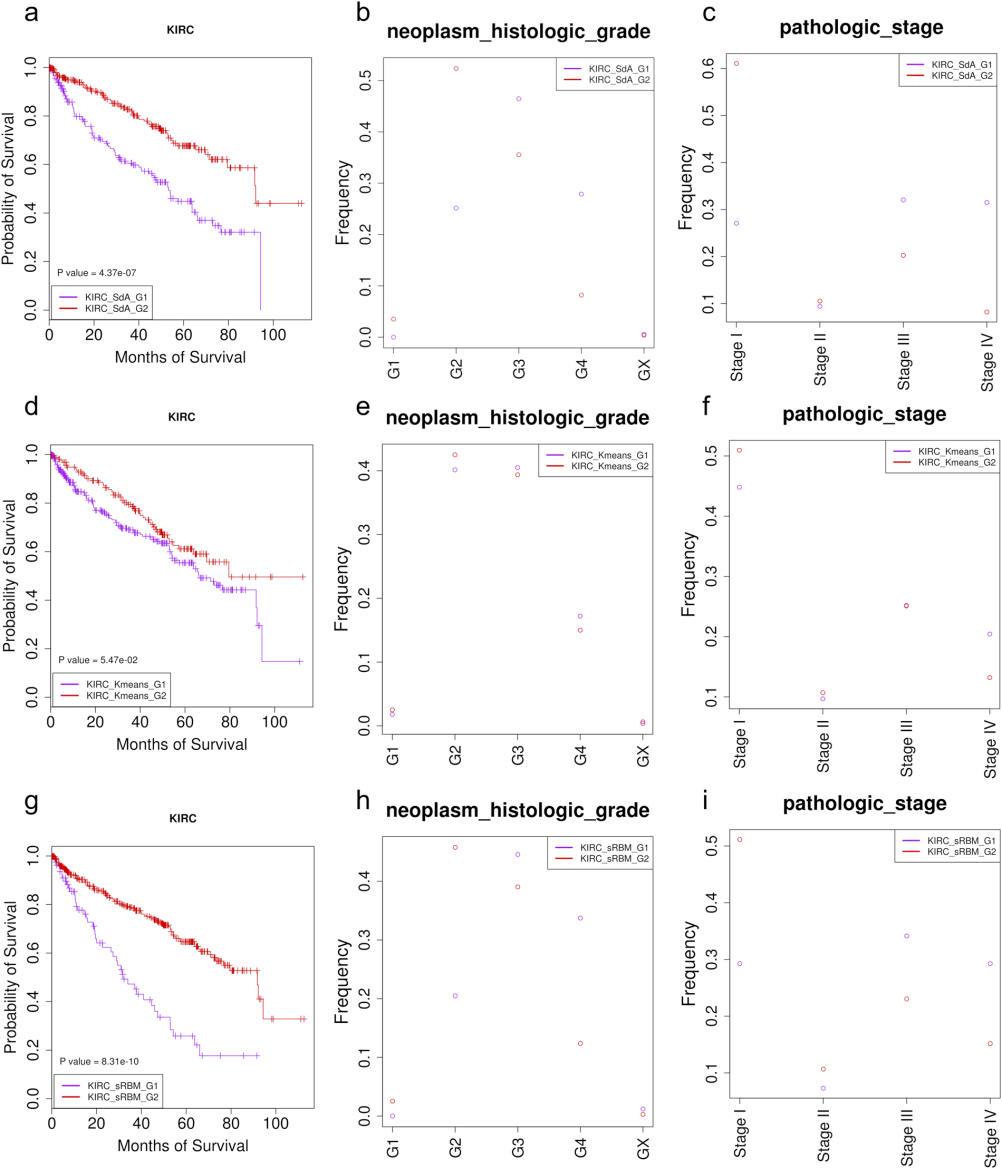
The clinical features for two subgroups of KIRC patients identified by three different methods (SdA, K-means and sRBM). (**a**–**c**) Are the survival probability, the neoplasm histologic grade distribution and the pathologic stage distribution between KIRC_SdA_G1 and KIRC_SdA_G2 for the SdA method. There are four grades for the neoplasm histology for KIRC patients: G1, G2, G3 and G4. GX is the cancer tissue that cannot be assessed. The higher grade of the cancer, the more abnormality of the cancer. There are four pathologic stages for KIRC patients: Stage I, Stage II, Stage III and Stage IV. The higher stage of the cancer, the more spread of the cancer. (**d**–**f**) Are the survival probability, the neoplasm histologic grade distribution and the pathologic stage distribution between KIRC_Kmeans_G1 and KIRC_Kmeans_G2 for the K-means method. (**g**–**i**) Are the survival probability, the neoplasm histologic grade distribution and the pathologic stage distribution between KIRC_sRBM_G1 and KIRC_sRBM_G2 for the sRBM method.

The probability of survival for the two KIRC group patients is significantly different (P value ~ 4.37e-07) (Fig. 3a). We observed the same phenomena for the neoplasm histologic grade (P value ~ 3.56e-12) and pathologic stage (P value ~ 2.68e-14) (Fig. 3b,c). The KIRC_SdA_G1 patients have significantly lower probability of survival time compared to the KIRC_SdA_G2 patients (Fig. 3a). There were much more G4 patients in KIRC_SdA_G1 while there were much more G2 patients in the KIRC_SdA_G2 group (Fig. 3b). Similarly, there were much more stage III and stage IV patients in KIRC_SdA_G1 while there were much more stage I patients in KIRC_SdA_G2 (Fig. 3c). The results indicate the SdA method works successfully on subtyping the KIRC patients, and the KIRC_SdA_G1 patients have much severer cancer progress and appropriate treatments should be developed differently for the two groups of patients.

### Differential expressed (DE) genes were identified between the KIRC_SdA_G1 and KIRC_SdA_G2 patients

Differential expression analysis was conducted between the two groups of patients using edgeR^14^. The significantly DE genes were called at thresholds of FDR 0.05 and fold change >2. A total of 634 DE genes were identified and most of them (495; ~78%) were down regulated in the KIRC_SdA_G2 patients (Supplementary Table 2). We further performed pathway enrichment analysis to test the function of the DE genes using the Reactome database (version 67) and its online tool (https://reactome.org/). Twenty terms were found at threshold of FDR less than 0.1 (Table 1). The terms were either associated with extracellular matrix (ECM) (such as, ECM organization, formation and degradation, etc), or cell cycle (such as, G2 arrest and G2/M transition, etc) or important signaling pathways (such as, regulation of IGF transport and updake by IGF binding proteins, post-translational protein phosphorylation, RHO GTPases activate IQGAPs and regulation of TLR by endogenous ligand, etc). The majority of the terms were linked with cancer development, especially metastasis. For example, ECM is a component of all mammalian tissues and a complex network of macromolecules that not only serves as the scaffold of an organ, but also involves in cell proliferation, migration, invasion, the onset of angiogenesis, or the resistance to apoptotic stimuli^15^. ECM plays a crucial role in cancer development^16^ and influences every cancer hallmark defined by Hanahan and Weinberg^17^. The cancer metastatic cascade is also critically influenced by ECM components^15^. ECM is highly dynamic and largely regulated by matrix metalloproteinases (MMPs)^18^. Several MMPs were in the DE gene set: MMP1, MMP7, MMP9 and MMP17. Two of them, MMP7 and MMP9, were reported that higher expression of the two genes with poor prognosis of ccRCC^19,20^, supporting our results that higher expression of the two genes with poor survival probability, higher neoplasm histologic grade and pathologic stage. The results from the pathway analysis were consistent with the clinical features of the two groups. In summary, our results implied that gene expression contributes to stratify the patients and the DE genes are potential biomarkers for separating the patients and are potential targets for developing appropriate treatments.

**Table 1:** Pathway enrichment analysis results for the significantly differential expressed genes between KIRC_SdA_G1 and KIRC_SdA_G2.

Pathway name	#Customer Input Genes	P Value	FDR
Polo-like kinase mediated events	12	1.64E-03	5.28E-09
Extracellular matrix organization	45	2.34E-02	7.86E-09
Regulation of Insulin-like Growth Factor (IGF) transport and uptake by Insulin-like Growth Factor Binding Proteins (IGFBPs)	25	9.03E-03	2.68E-08
Collagen formation	22	7.40E-03	5.39E-08
Degradation of the extracellular matrix	24	1.05E-02	1.53E-06
Post-translational protein phosphorylation	20	7.75E-03	1.86E-06
Metallothioneins bind metals	8	1.14E-03	2.57E-06
Assembly of collagen fibrils and other multimeric structures	15	4.77E-03	3.53E-06
Response to metal ions	8	1.49E-03	1.80E-05
Activation of Matrix Metalloproteinases	10	2.49E-03	1.97E-05
Collagen biosynthesis and modifying enzymes	14	5.41E-03	6.04E-05
Collagen degradation	13	4.91E-03	8.78E-05
Resolution of Sister Chromatid Cohesion	18	9.53E-03	3.01E-04
Insulin-like Growth Factor-2 mRNA Binding Proteins (IGF2BPs/IMPs/VICKZs) bind RNA	5	9.25E-04	6.54E-04
RHO GTPases activate IQGAPs	8	2.56E-03	6.87E-04
TP53 Regulates Transcription of Genes Involved in G2 Cell Cycle Arrest	6	1.49E-03	9.00E-04
G2/M Transition	23	1.51E-02	9.20E-04
Mitotic G2-G2/M phases	23	1.52E-02	1.04E-03
Regulation of TLR by endogenous ligand	7	2.20E-03	1.34E-03
Cell Cycle, Mitotic	47	4.05E-02	1.44E-03

These terms formed three major groups: terms associated with ECM, terms associated with cell cycle, and terms associated with other important signaling pathways.

### DE analysis on miRNAs and proteins between the KIRC_SdA_G1 and KIRC_SdA_G2 patients

Similar DE analysis was performed for miRNAs using edgeR^14^ and 24 DE miRNAs were found at thresholds of FDR at 0.05 and fold change >2 (Supplementary Table 3). The DE miRNA with the lowest q value (p value after multiple testing correction using Benjamini & Hochberg method), hsa-mir-139, has been shown in multiple studies that low expression of hsa-mir-139 links to the advance stages of ccRCC^21,22^. In our results, hsa-mir-139 was low expressed in KIRC_SdA_G1, the groups of patients with poor survival probability, higher cancer stage and grade. The results were consistent with the existing publications, demonstrating that miRNAs can be potential biomarkers for diagnosis and treatments.

T-test was employed for the protein DE analysis and the significantly DE proteins were called at thresholds of FDR at 0.05 and fold change >2. One DE protein was found, SERPINE1, which is a member of the serine protease inhibitor sup-family that negatively regulates fibrinolysis and impairs the dissolution of clots (Supplementary Table 4). It also regulates cell migration that is independent of its function as a protease inhibitor reported in GeneCards (https://www.genecards.org/cgi-bin/carddisp.pl?gene=SERPINE1). SERPINE1 was more than two-fold higher expressed in the KIRC_SdA_G1 patients, which had lower probability of survival and higher neoplasm histologic grade and pathologic stage. Our results were supported by the data in Human Protein Atlas database that higher expression of SERPINE1 is associated with lower probability of survival in multiple cancers: urothelial cancer, stomach cancer, lung cancer, head and neck cancer, colorectal cancer and cervical cancer, demonstrating that SERPINE1 is a high confident protein biomarker (https://www.proteinatlas.org/ENSG00000106366-SERPINE1/pathology).

### Comparison with K-means clustering method

To test whether the deep learning method is more accurate and effective than other methods, the commonly used method, K-means clustering, was examined using the R package, K-means. We did two-means clustering multiple times with different initial centroids, and then did the same survival analysis for the two groups identified by K-means. We found that K-means cannot significantly separate the patients at threshold of p value at 0.05, although the p value was close to 0.05 (P value for survival analysis is ~0.055; Fig. 3d). Further, we compared between the two K-means clusters for the neoplasm histology and pathology (Fig. 3e,f). We did not obtain significantly differences between the two clusters, although we found similar frequency patterns across different grades of neoplasm histology and different stages of pathology. The results suggest that SdA method is a more promising method on classifying patients given complicated datasets.

### Comparison with the stacked of Restricted Boltzmann Machines (sRBM) method

Several studies showed that sRBM works on cancer sample stratification. We used the same layer structures for the sRBM as the SdA method: one hidden layer with 50 units for miRNA expression and protein expression, two hidden layers with 500 and 50 units each for gene expression, methylation and CNA, three layers with 50, 10 and 1 unit/units each for the joint hidden layers (Fig. 1a). Since the speed to run one round of two-fold cross validation using sRBM was much slower, we did not test multiple parameters in different ranges but ran sRBM using the parameters selected for the SdA approach, that were learning rate at 0.01, batch size at 50 and the epoch at 5000, except the number of persistent chains was tested at 5, 10 and 30. For the persistent chain parameter at 10 and 30, the correlation between the input and the LJHL reconstructed input for protein dataset was less than 0, thus, we discarded this result and chose the results from the persistent chain parameter at 5 that all the correlations were larger than 0. The correlations between the original input and the reconstructed input from the LIHL and LJHL were generally much lower than the SdA method (Supplementary Fig. 1a,b).

We used K-means to separate the hidden values into two groups, named KIRC_sRBM_G1 and KIRC_sRBM_G2. The survival probability was significantly different between KIRC_sRBM_G1 and KIRC_sRBM_G2 (p value ~ 8.31e-10) and KIRC_sRBM_G1 patients had much less probability of survival, similar to the results from the SdA method (Fig. 3g). There was also significant difference between the two groups for the pathologic stage (p value ~ 8.51e-07) and neoplasm histologic grade (p value ~ 0.00041) with similar distribution of the frequency for each pathologic stage and for each neoplasm histologic grade to SdA that the sRBM_G1 group contained much more patents with higher pathologic stages or neoplasm histologic grades (Fig. 3g–i), demonstrating the consistency between the sRBM and SdA methods.

### Validation and application of the model learned from KIRC dataset (KIRC model) using/on LUAD and LGG datasets

**Figure 4:**
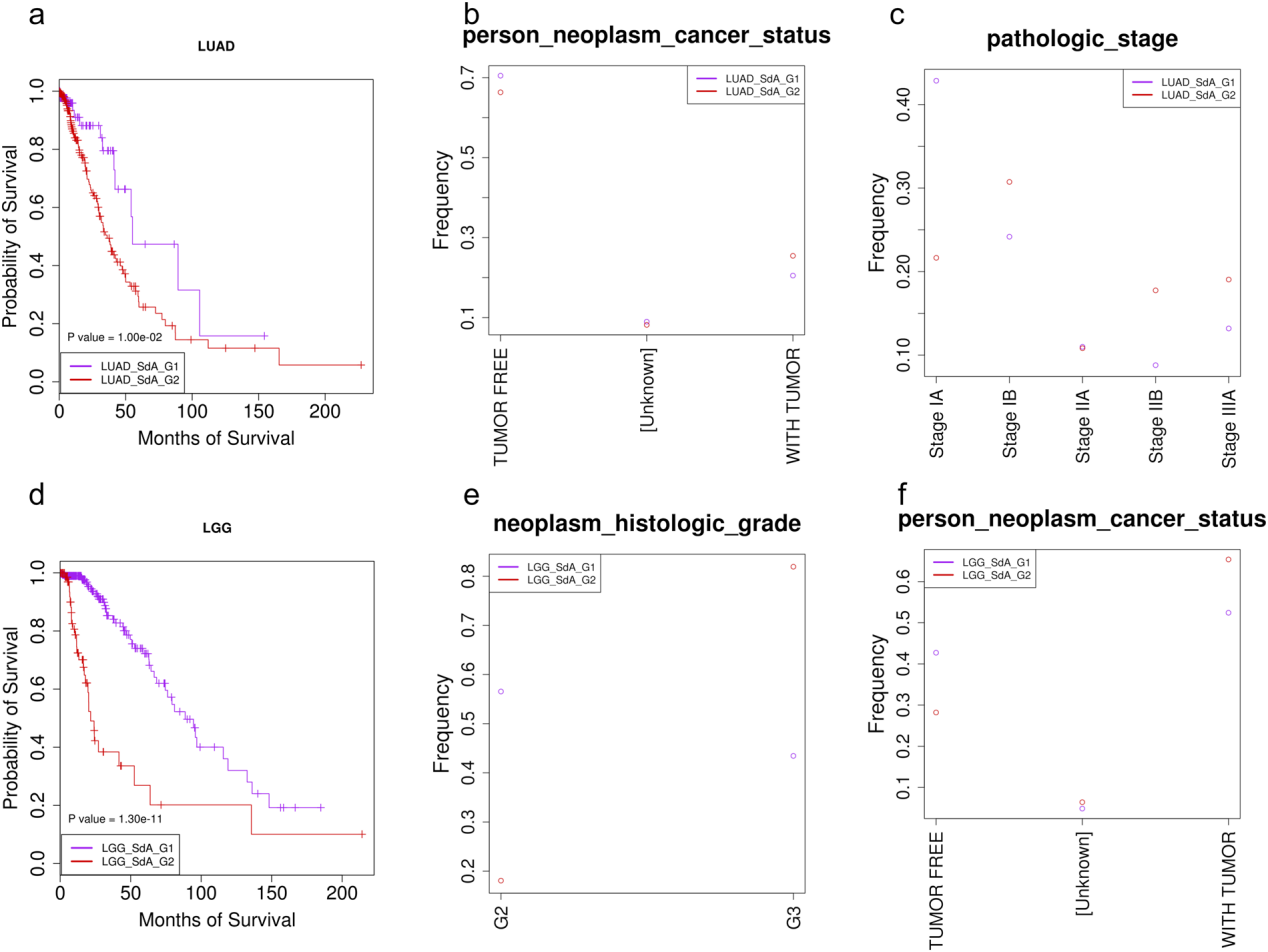
The clinical features for the two subgroups of LUAD and LGG patients. (**a**–**c**) Are the survival probability, the neoplasm cancer status distribution and the pathologic stage distribution between LUAD_SdA_G1 and LUAD_SdA_G2 for the LUAD patients. (**d**,**e**,**f**) Are the survival probability, the neoplasm histologic grade distribution and the neoplasm cancer status distribution between LGG_SdA_G1 and LGG_SdA_G2 for the LGG patients. There is no neoplasm histologic grade feature in TCGA for the LUAD patients, but two person neoplasm cancer status are recorded in TCGA: TUMOR FREE and WITH TUMOR. For the LGG patients, there is no pathologic stage feature annotated in TCGA, therefore, we included the neoplasm cancer status distribution feature in the analysis.

To further validate the SdA method and also show its general application, we applied the KIRC model as a pre-trained model to classify LUAD and LGG patients, two independent datasets. We first downloaded the miRNA, protein and gene expression, methylation and CNA datasets from TCGA for LUAD and LGG patients and processed the data the same way as the KIRC dataset. Since the dataset and the goal of the work were similar between KIRC, LUAD and LGG, transferring learning probably works and produces better results than learning directly from LUAD and LGG datasets separately. We transferred the hidden variable values from KIRC model to LUAD and LGG models and then fine-tuned the variables using LUAD and LGG datasets separately with a lower learning rate (0.0005). The methods split the patients into two clinical relevant groups for LUAD and LGG: the LUAD_SdA_G2 group has significantly lower survival probability than the other group (Fig. 4a), similar phenomena was observed for LGG patients (Fig. 4d). No neoplasm histologic grades were found in TCGA for LUAD patients, but there were two neoplasm cancer status for LUAD and LGG: TUMOR FREE and WITH TUMOR. LUAD_SdA_G2 had much less patients for the status of TUMOR FREE than LUAD_SdA_G1 and more patients for the status of WITH TUMOR (Fig. 4b), similar results were obtained for LGG (Fig. 4f). Five pathology stages/sub-stages had more than 5 patients, which were included in the analysis. LUAD_SdA_G2 had much less patients in Stage IA and more patients in advance stages of Stage IB, IIB and IIIA than LUAD_SdA_G1 (Fig. 4c). No pathology stages were found in TCGA for LGG patients, but neoplasm histologic grades were available. We found much more LGG_SdA_G1 patients at grade G2 than LGG_SdA_G2, while much more LGG_SdA_G2 patients were found at grade G3 than LGG_SdA_G1 (Fig. 4e). The three clinical associated features were consistent with each other for two independent datasets, indicating that the SdA method and the model learned from KIRC dataset is able to integrate multi-platform datasets to subtype cancer patients and most likely can apply to other similar tasks.

## Discussion

In this work, we developed a SdA model to classify the kidney clear cell cancer patients based on multi-platform genomic datasets. The model classified the patients into two groups. The KIRC_SdA_G1 group had significantly lower survival probability, higher grade of neoplasm histology and pathology than the KIRC_SdA_G2 group. We compared the SdA model with K-means clustering method. SdA produced a much clearer classification of the KIRC patients according to the clinical information, indicating SdA is a more promising method for cancer subtype discovery. We did limited tests using RBM based DBN model and obtained similar results as the SdA method. Further, we identified a group of DE genes, miRNAs and one protein that may be potential biomarkers for developing novel diagnosis method and personalized treatments. In the end, we demonstrated using two independent datasets that the SdA method and the learned models can apply to other similar tasks.

In our current study, we did not consider the biological connections between each dataset, but treated each dataset independently in the integration analysis. Then we performed the differential analysis for gene, miRNA and protein expression separately between the two subgroups. However, it is widely accepted that miRNAs and methylation typically repress gene expression both at mRNA and protein level. It may improve the accuracy for biomarker discovery by including the biological connections between the datasets and give a better explanation on the role of biomarker in cancer development.

We compared our subtyping results with a previous study from TCGA consortium (Chen *et al*.)^23^, in which RCC was classified into nine subtypes based on the same types of genomic datasets, including three subtypes (CC-e.1, CC-e.2 and CC-e.3) of ccRCC that have better (CC-e.2), worse (CC-e.3), and intermediate (CC-e.1) patient survival probablity. All the 441 ccRCC patients used in our study were included in the Chen *et al*.^23^ study. Of these 441 patients, ~94% were mapped to CC-e.1, CC-e.2 and CC-e.3. In our study, KIRC_SdA_G1 contained 174 patients and was the group with worse survival probability. The majority of the KIRC_SdA_G1 patients were in the subtypes that defined as worse and intermediate patient survival categories (CC-e.3: ~50% and CC-e.1: ~28%) in the Chen et al. study. (Supplementary Table 5) Similarly, most of the KIRC_SdA_G2 patients were in the subtype of CC-e.2 (~68%), indicating the consistency between our study and the Chen *et al*. study.

One drawback of the deep learning methods is overfitting. We used three approaches to control the overfitting. First, we added the correlation between the reconstructed input and the original input as a standard in model parameter selecting process that is independent to the cross entropy used in the training process. Second, we separated the whole datasets into two subgroups with one used for training and the other used for testing. The parameters were selected based on the correlations that were the top 10 highest correlations both for the training and test datasets. Third, we selected the final set of parameters that had the largest corruption level. With the three approaches, we were inclined to produce a more robust model.

Although an autoencoder is a deterministic model, dA is a stochastic model that corresponds to a generative model^24^. It showed that SdA is systematically better than stacked autoencoder (SA)^25^ but in special cases, SA is better or comparable to SdA^26^. A nice property of dA is that it naturally handles missing values or noising values or multi-modal values because of being trained using corrupted input. RBM is also a stochastic model, similar as dA. Although dA and RBM are different in training process and formula format, they function in a similar way (mapping the inputs to hidden representations) (Fig. 1). SdA and sRBM have been used on similar tasks, such as classification and feature extraction. According to our and others’ research, there is no clarification whether SdA is better than sRBM or not on classification with current algorithms^26,27^. For some cases, the SdA may be better than sRBM, such as, for the input data highly corrupted in these studies^26,28^ and in speech recognition^29^. For some cases, the sRBM may be better than SdA, such as in Tan and Eswaran’s study^30^. Nevertheless, SdA and sRBM are generally comparable to each other. In our study, we observed similar separation on the KIRC patients using sRBM method and p value for the survival probability between the two subtypes were comparable to each other, although the p value for the neoplasm histologic grades and pathologic stages were much smaller for SdA method. Since we did not test a wide range of the parameters for sRBM as training the SdA model, we cannot conclude that in our case, SdA works better than sRBM. But the consistent results between the SdA and sRBM method showed that the deep learning method is able to integrate multi-platform to stratify the patients into subgroups.

Although in comparison with shadow deep learning method, K-means can produce comparable or even better results^31^, SdA and sRBM behaves better than K-means on classifying using complicated datasets. In our study, K-means was able to separate the patients into two groups, but the groups were not significantly different on survival time. Because K-means is sensitive to the initial assignment of the centroids, we tested several initializations of the centroids, but still obtained similar results. Another study^13^ also reported that sRBM works better than K-means. These results suggest that comparing with the simple method, K-means, the multi-layer deep learning method is much more robust on dealing with multi-platform and complicated datasets.

It is common that deep learning methods consume more time to train the model than the commonly used methods, such as K-means and PCA. However, as shown in our study, the SdA method performed apparently better than K-means. To reduce the computing time on generating optimal parameters for SdA models and learning from scratch, we tested whether we can use the learned model as a pre-trained model. As expected, the KIRC model worked efficiently on classifying the LUAD and LGG patients by taking it as a pre-train model. The limitation of transfer learning is that the model probably cannot be used directly on tasks with less similarity. When it occurs, a new model will be needed to learn from scratch.

## Materials and Methods

### Process of the KIRC dataset

miRNA, protein and gene expression, methylation and CNA were downloaded and re-formatted using TCGA-assembler^32^ for KIRC patients. TCGA-assembler processed each type of data into a matrix that makes easier for downstream analysis. Some data from the same patient were duplicated either because multiple samples were collected from the same patient, or different portions of the sample were measured or sequenced in different orders or different plates. The average value of the duplicated data points was used in the downstream analysis.

For the gene expression and miRNA expression, both the counts and FPKM (Fragments Per Kilobase of transcript per Million mapped reads) values were downloaded. The FPKM values were used for classification and the counts were used for differential expression analysis. The normalized protein expression was downloaded and used both for the classification and differential expression analysis.

The Infinium Human Methylation 27 BeadChip and Infinium Human Methylation 450 BeadChip were downloaded, processed and merged by TCGA-assembler^32^. During the merge process, the data were normalized using quantile method.

The CNA data without the probes frequently containing germline CNVs were downloaded and processed using TCGA-assembler^32^. A data matrix was produced with rows corresponding to CNAs and columns corresponding to samples. The process procedure performed by TCGA-assembler mapped the CNA locations to genes. Some genes are within the same CNA region, therefore, duplicated rows in the data matrix were produced. We removed the duplicated rows from downstream analysis.

We filtered the patients with missing values for any of the five datasets. In the end, we obtained a total of 441 patients for each dataset. Before fit into the deep learning methods, all the input values were normalized across samples to a value between 0 and 1 using the formula of

1 $$\frac{x-{x}_{min}}{{x}_{max}-{x}_{min}+a},$$

where *x* is the input value, *x*_*min*_ is minimum value across all the samples, *x*_*max*_ is the maximum value across all the samples and *a* is a small number to avoid potential errors in the calculation.

### Process of the LUAD and LGG datasets

The miRNA, protein and gene expression, methylation and CNA datasets for LUAD and LGG patients were downloaded and processed in the same way as the KIRC dataset. The sample size for LUAD is much smaller, comparing to KIRC dataset, and a total of 354 patients were remained. The LGG dataset do not have methylation data from Infinium Human Methylation 27 BeadChip, therefore, a similar number of methylated loci from Infinium Human Methylation 450 BeadChip was used. All the data were normalized to a value between 0 and 1 in the same way as done for the KIRC dataset (formula 1).

### Stacked denoising autoencoders (SdA) method

A denoising autoencoder (dA) is an extension of an autoencoder. An autoencoder is a variant of neural networks that commonly used for feature extraction. It maps the input data into a hidden representation through a deterministic function. When the hidden units are less than the input units, the input is compressed. The training objects for an autoencoder is to build the compact features that can reconstruct the input data. By minimizing the difference between the reconstructed input and the original input, the intrinsic features of the data are learned. Therefore, an autoencoder is an un-supervised method that is independent of the data labels. Denoising autoencoders improve the basic autoencoders by corrupting part of the input data. By learning from the corrupted input, dA could not only capture the essential features of the input robustly, but also avoid the problem of reconstructing the same input from an un-corrupted input. Since dA reconstructs the input from the corrupted input that involved in sampling, dA becomes a stochastic model. Normally, a simple dA contains three layers: an input data layer, a hidden layer and a reconstructed input layer. And the input data is corrupted. Here we used binomial noise to corrupt the input, that is part of the input data, *x*, is randomly altered into 0, called *x*^*T*^. The hidden layer (*y*) was built based on formula 2, where *W* is the weight matrix, *b* is the bias term, and *s* is the sigmoid function. The reconstructed input layer (*z*) was built based on the formula 3, where *W*′ is a tied weight of *W* and *b*′ is the bias term. Cross-entropy (formula 4) was used to measure the difference between the input data and the reconstructed input data. The training process is to find the *W*, *W*′, *b*, *b*′ that can minimize the cross-entropy.

2 $$y=s(W{x}^{T}+b),$$

3 $$z=s(W^{\prime} y+b^{\prime} ),$$

4 $$L(x,z)=-\,\mathop{\sum }\limits_{k=1}^{m}\,[{x}_{k}\,log\,{z}_{k}+(1-{x}_{k})\,\log \,(1-{z}_{k})].$$

In our study, we have five types of genomic data, miRNA expression, protein expression, gene expression, methylation and CNA. To subgroup the patients based on the five genomic datasets, we developed stacked layers of dA (SdA). Each dataset has its independent hidden layers and then the hidden layers from the five datasets were combined together (Fig. 1a). The size of miRNA and protein expression datasets is much smaller, therefore, one hidden layer with 50 units was used. For the gene expression, methylation and CNA, two hidden layers with 500 and 50 units were used. Thus, the final hidden layer for each dataset contains 50 units that were combined to form the top joint layers (Fig. 1a). The top joint layers contain three layers with 50, 10 and 1 unit/units separately. For each hidden layer, we trained separately to get the optimal output for the input of next layer. The hidden values produced from the last joint hidden layer using formula (2) were clustered into two groups using K-means. We adapted and modified the programs developed by Yifeng Li^33^ for the SdA analysis.

To train the model, we tested a wide range for the following parameters: the learning rate at 0.005, 0.01, 0.05, 0.1, 0.15, 0.2, 0.3 and 0.5; the corruption level at 0.01, 0.05, 0.1, 0.2 and 0.5; the batch size at 10, 20, 50 and 100; and the epoch size at 1000, 2000 and 5000. We did two-fold cross validation and calculated the correlations between the reconstructed input from the last joint hidden layer and the last individual hidden layer with the original input from both the training and test datasets (Fig. 1b). We summed the correlations from each dataset and selected 10 sets of parameters for the training and test datasets that can produce the top 10 highest summed correlations. The parameters present in both the training and the test datasets were retained (Fig. 1b). We then selected the one with the highest corruption level as the final parameters which are leaning rate at 0.01, corruption level at 0.2, batch size at 50 and the epoch at 5000.

### Stacked of restricted boltzmann machines (sRBM) method

Deep Belief Networks (DBN) normally refers to stacked of RBMs. RBM, unlike the deterministic model of the autoencoder method, is a generative model, using stochastic process to map the input to hidden variables, thus similar to dA. Several papers showed DBN works on cancer subtype discovery^12,13^. We used the same hidden layer structures to perform the analysis (Fig. 1a). The algorithm of contrastive divergence was used to learn the parameters. We did not test the parameters at different ranges since the running time for training the sRBM is much longer than the SdA method. Here, we chose the same parameters as we used in the SdA: the learning rate at 0.01, the batch size at 50 and the epoch size at 5000. Since the persistent chains is not in the SdA method, we tested several values for the number of persistent chains: 5, 10 and 30. We adapted and modified the programs developed by Yifeng Li^33^ for the sRBM analysis.

### Differential analysis (DE) for gene expression, protein expression and miRNA expression

edgeR^14^ was used for the DE gene and miRNA analysis between the KIRC_SdA_G1 and KIRC_SdA_G2 patients. Upperquartile from edgeR was used to normalize the raw counts across samples. The genes and miRNAs with expression less than 1 Counts Per Million (CPM) in at least half of the SdA_G1 samples were removed from the analysis. The final set of DE genes and miRNAs were called at thresholds of FDR at 0.05 and fold change >2. The protein differential expression analysis was performed using two-tailed t-test and the significantly DE proteins were called at thresholds of FDR at 0.05 and fold change >2.

### Validation and application of the model learned from KIRC dataset (KIRC model) using/on the LUAD and LGG datasets

We downloaded the miRNA, protein and gene expression, methylation and CNA datasets from TCGA for LUAD and LGG patients and processed the same way as KIRC. To save the time on selecting the optimal model parameters, we used the KIRC model as a pre-trained model and do fine-tuning using the LUAD and LGG datasets (also called transferring learning). Since the three datasets (KIRC, LUAD and LGG) have similar data structure and goal of subtyping, transferring learning most likely can produce better results than learning from LUAD and LGG directly^34^. To do transferring learning, we initialized the variables of the LUAD and LGG models with the learned variable values from KIRC model, and then fine-tuned the variables using the LUAD and LGG datasets separately with a lower learning rate at 0.0005, 20 fold less than the KIRC model as suggested in the study of^35^.

## Supplementary information

Supplementary Information

Supplementary Tables 1-5

## Data Availability

The data are available along with the manuscript.
