# Author Correction: Integrating multi-platform genomic datasets for kidney renal clear cell carcinoma subtyping using stacked denoising autoencoders

**DOI:** 10.1038/s41598-019-55817-0

**Published:** 2019-12-19

**Authors:** Tongjun Gu, Xiwu Zhao

**Affiliations:** 1 Bioinformatics, Interdisciplinary Center for Biotechnology Research, University of Florida, Gainesville, FL USA; 2 Department of Ophthalmology & Visual Sciences, University of Michigan, Ann Arbor, MI USA

Correction to: *Scientific Reports* 10.1038/s41598-019-53048-x, published online 13 November 2019

The Acknowledgements section in this Article is incomplete.

“We thank Dr. Xiaolin Li and Yanjun Li from University of Florida for their suggestions on the method used in our study.”

should read:

“We thank Dr. Xiaolin Li and Yanjun Li from University of Florida for their suggestions on the method used in our study. Publication of this article was funded in part by the University of Florida Open Access Publishing Fund.”

