# Supplementary material for: Integrating multi-platform genomic datasets for kidney renal clear cell carcinoma subtyping using stacked denoising autoencoders
Source: Sci Rep. 2019 Nov 13;9:16668. doi: 10.1038/s41598-019-53048-x (PMC6853929; doi:10.1038/s41598-019-53048-x)
Supplement: Supplementary file 1 — Supplementary Information [file 41598_2019_53048_MOESM1_ESM.pdf]

Integrating multi-platform genomic datasets for kidney renal clear cell carcinoma subtyping using stacked denoising autoencoders

Tongjun Gu[1*], Xiwu Zhao[2*]

[1]Bioinformatics, Interdisciplinary Center for Biotechnology Research, University of Florida, Gainesville, FL, USA
[2] Department of Ophthalmology & Visual Sciences, University of Michigan, Ann Arbor, MI, USA

Co-correspondence:

Tongjun Gu
2033 Mowry Road, Gainesville, FL 32610
Phone: (352) 273-8058
Fax: (352)273-8070
Email: tgu@ufl.edu

Xiwu Zhao
1000 Wall Street, Ann Arbor, MI 48105
Phone: (734)-936-8548
Fax: (734)-936-7231
Email: xiwuzhao@umich.edu

Legends for supplementary figures

Supplementary Figure 1. Pearson correlation between the KIRC original input and the reconstructed input from the LIHL and LJHL for the sRBM method with different number of persistent chains. a, the correlation from the LIHL. b, the correlation from the LJHL. Y axis is the correlation and x axis is the five datasets: miRNA expression, protein expression, gene expression, methylation and CNA. K_5, K_10 and K_30 represent the number of persistent chains at 5, 10 and 30 respectively.

Legends for supplementary tables

Supplementary Table 1. The subtypes and clinical information for each patient.
Supplementary Table 2. Differential expressed genes between KIRC_SdA_G1 and KIRC_SdA_G2.
Supplementary Table 3. Differential expressed miRNAs between KIRC_SdA_G1 and KIRC_SdA_G2.
Supplementary Table 4. Differential expressed proteins between KIRC_SdA_G1 and KIRC_SdA_G2.
Supplementary Table 5. Comparison with Chen et al's study.

Supplementary Figures

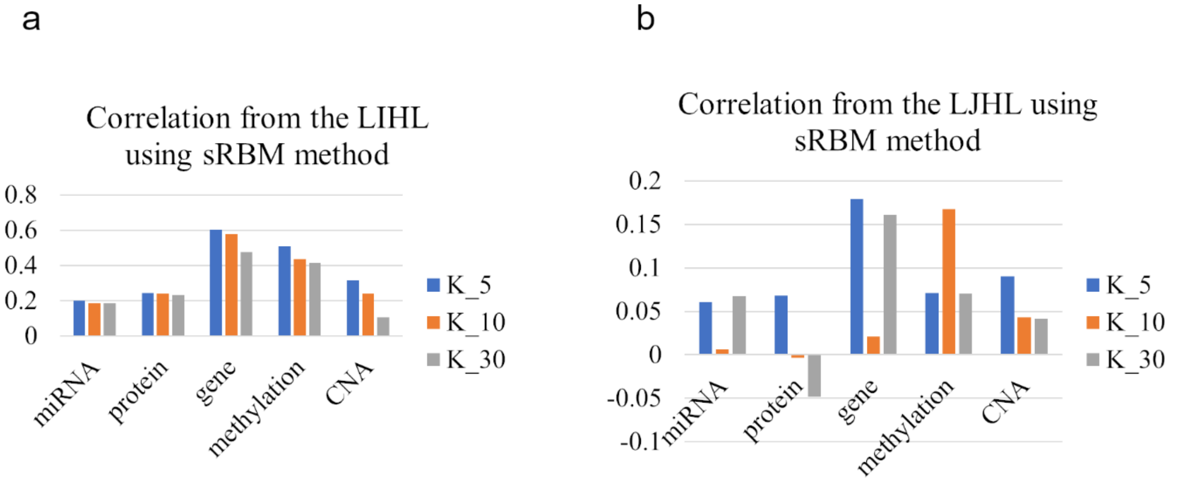

Supplementary Figure 1. Pearson correlation between the KIRC original input and the reconstructed input from the LIHL and LJHL for the sRBM method with different number of persistent chains. a, the correlation from the LIHL. b, the correlation from the LJHL. Y axis is the correlation and x axis is the five datasets: miRNA expression, protein expression, gene expression, methylation and CNA. K_5, K_10 and K_30 represent the number of persistent chains at 5, 10 and 30 respectively.
